# Association of the Chromosome Replication Initiator DnaA with the *Escherichia coli* Inner Membrane *In Vivo*: Quantity and Mode of Binding

**DOI:** 10.1371/journal.pone.0036441

**Published:** 2012-05-04

**Authors:** Tomer Regev, Nadav Myers, Raz Zarivach, Itzhak Fishov

**Affiliations:** 1 Department of Life Sciences, Ben Gurion University of the Negev, Beer-Sheva, Israel; 2 National Institute of Biotechnology, Ben-Gurion University of the Negev, Beer-Sheva, Israel; Indian Institute of Science, India

## Abstract

DnaA initiates chromosome replication in most known bacteria and its activity is controlled so that this event occurs only once every cell division cycle. ATP in the active ATP-DnaA is hydrolyzed after initiation and the resulting ADP is replaced with ATP on the verge of the next initiation. Two putative recycling mechanisms depend on the binding of DnaA either to the membrane or to specific chromosomal sites, promoting nucleotide dissociation. While there is no doubt that DnaA interacts with artificial membranes *in vitro*, it is still controversial as to whether it binds the cytoplasmic membrane *in vivo*. In this work we looked for DnaA-membrane interaction in *E. coli* cells by employing cell fractionation with both native and fluorescent DnaA hybrids. We show that about 10% of cellular DnaA is reproducibly membrane-associated. This small fraction might be physiologically significant and represent the free DnaA available for initiation, rather than the vast majority bound to the *datA* reservoir. Using the combination of mCherry with a variety of DnaA fragments, we demonstrate that the membrane binding function is delocalized on the surface of the protein’s domain III, rather than confined to a particular sequence. We propose a new binding-bending mechanism to explain the membrane-induced nucleotide release from DnaA. This mechanism would be fundamental to the initiation of replication.

## Introduction

DnaA is a ubiquitous protein which initiates chromosome replication in bacteria and its activity in initiation is restricted to once per cell division cycle. DnaA binds to distinct high-affinity sites on the origin of replication, *oriC*, called DnaA-boxes, and to other sites on the chromosome with lower affinity. Concerted binding to *oriC* sites forces unwinding of the adjacent AT-rich region with the consequent opening of the double helix. Recruitment of DnaB helicase by DnaA and assembly of the replisome effectively initiates the DNA replication process [1]–[4].

*E. coli* DnaA is 467 residues long with a molecular weight of ∼52 kDa and is composed of four distinct domains [1], [5]. There is no crystal structure available for the full DnaA from *E. coli* although structures of the first (residues 1–108) and the last (residues 374–467) domains have been determined [6], [7]. The N-terminus of DnaA is responsible for protein-protein interactions and oligomerization [8], [9] and for DnaA-DnaB interaction [10]. Domain II is variable in size among bacteria, alternating between 6 to 247 residues and its function is rather unclear. In *E. coli*, deletion of domain II (amino acids 87–134) showed it was not essential for cell viability [5] although it was recently shown to participate in regulation of replication initiation [11]. This expendability of domain II and its being virtually unfolded has been exploited by [12], [13] and in this work to insert a gene encoding a fluorescent protein into the DnaA sequence so as to obtain an active fluorescent hybrid. Domains III and IV of DnaA from *Aquifex aeolicus* (35% identity and 65% similarity with DnaA from *E. coli*) was the first structure that was determined [14]. Domain III belongs to the AAA^+^ ATPases family and is responsible for ADP/ATP binding [15]. Domain IV is responsible for DnaA-DNA interaction, binding to the major groove of DNA via a helix-turn-helix motif [7].

Initiation of chromosome replication by DnaA is a highly regulated event in bacteria since the timing of DNA replication is crucial for steady-state cell growth. *E. coli* cells growing at doubling times shorter than the replication time must initiate new rounds of chromosome replication prior to the completion of the previous one, leading to a state in which multiple copies of the *oriC* exist inside the cell [16]. All copies of *oriC* are initiated synchronously revealing the existence of a tight regulation [17]. The initiation process is regulated by at least three known mechanisms: control of origin availability mediated by SeqA sequestration of hemi-methylated GATC sequences [18], control of accessibility - titration of DnaA at the datA locus [19] and direct activation/inactivation of DnaA [20]. The last of these mechanisms is driven by ATP binding and hydrolysis with only the ATP-DnaA form being functional in initiation [21]. Its inactivation component is termed RIDA (replication inactivation of DnaA), promoting DnaA ATPase activity immediately after initiation [4]. Only towards the next initiation time is ADP-DnaA reactivated to the ATP form, starting a new replication cycle. *De novo* synthesis of DnaA in the ATP form is not sufficient to support new initiation cycles, and therefore, efficient recycling of ADP-DnaA is required [22]. Thus far, two recycling mechanisms promoting dissociation of ADP from DnaA and its subsequent replacement by ATP were suggested [23], [24]. Both presume that the binding of DnaA either to the membrane or to specific chromosomal sites induces a transition of the protein to a putative conformation with a lower nucleotide affinity.

The ability of phospholipids to activate purified DnaA was discovered in experiments with *in vitro* DNA replication [24], [25]. Specifically, acidic phospholipids in a fluid membrane appeared to be most effective [26]. Impaired initiation of replication in bacteria limited in acidic phospholipids synthesis suggested that phospholipid activation of DnaA may function also *in vivo* [27]. Intensive search for the membrane binding region pointed to the end of domain III adjacent to domain IV [28], [29]. The most common membrane binding structure searched for was an amphiphilic α-helix, and, indeed, several positively charged and hydrophobic residues in the tested region were found essential for phospholipid sensitivity of DnaA [30]–[33]. The switching role of DnaA-membrane binding was supported by a cooperative transition in nucleotide binding affinity driven by DnaA density on the membrane surface [34]. Moreover, DnaA mutant L366K, which rescued an acidic phospholipid-deficient strain from growth arrest [35], required less phospholipids for its activation [36]. These findings, together with the concept of membrane domains [37], [38], suggest the nature of the mechanism responsible for transducing continuous cell growth into DnaA activity switch at the appropriate time in the cell cycle.

Recently, a new rejuvenation pathway was discovered which is mediated by binding of DnaA to *E. coli* chromosomal regions DARS1 and DARS2 (DnaA-reactivating sequences) [23]. Formation of DnaA oligomers on these sites promoted the dissociation of ADP from DnaA *in vitro*. These DNA regions were shown to increase ATP-DnaA levels and affected initiation of replication *in vivo* [23]. Deletion of both DARS regions resulted in cells with an essentially decreased *oriC*/mass ratio [23], however, the viability of these cells implies the existence of an alternative mechanism for ATP-DnaA regeneration. Obviously, the DARS-dependent regulatory mechanism is responsive to the chromosome replication state, while its link to cellular physiology is yet unclear.

Intracellular localization of DnaA was first investigated by fractionation of *E. coli* cells showing a remarkable portion of DnaA associated with the membrane [39]. Later, this result was also reproduced with *H. pylori* and *M. tuberculosis* [40], [41], indicating that the phenomenon may be universal in the bacterial world. Immunolabeling of fixed cells revealed DnaA in the membrane vicinity [42]. A more detailed and physiologically accurate visualization of DnaA in life cells was achieved by the construction of DnaA-fluorescent protein hybrids [12], [13], [43]. In cells expressing these constructs, DnaA was located predominantly on the chromosome, visible as foci ascribed to *oriC* and *datA* loci or as a helical pattern. In contrast to the previous studies, the last investigations provided no conclusive evidence for DnaA-membrane localization. Along with the new DARS activation mechanism, this raises a question whether the long-standing membrane-promoted DnaA activation actually exists in live cells. To address this question, the first essential step is to verify localization of DnaA at the membrane *in vivo*.

In this work we sought DnaA-membrane interaction in *E. coli* cells employing cell fractionation with both native and fluorescent DnaA hybrids. For compatibility with previous results, the fluorescent protein mCherry was inserted at the same position into DnaA as in the work of the Crooke’s group [12]. The localization of fluorescent DnaA was followed microscopically and quantified in cell fractions. We demonstrate that a small but reproducible portion of DnaA is indeed membrane associated. Moreover, using a combination of mCherry with a variety of DnaA fragments, we argue that the membrane binding function cannot be ascribed to a specific amphipathic helix, but is rather delocalized at the protein surface. Based on the *E. coli* DnaA structure model, a binding-bending mechanism is suggested, explaining the membrane-induced nucleotide release from DnaA.

## Results

### 1. Distribution of Endogenous DnaA in Cell Fractions

**Figure 1:**
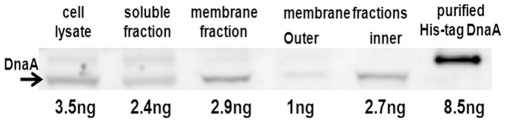
Immunoblot of cell fractions derived from wt *E. coli* BL21 (not containing any plasmids). A steady-state growing culture was disrupted using French-press and fractionated as described in Materials and Methods. The fractions were subjected to SDS-PAGE (10 µg of protein loaded per lane), western-blotted and exposed to anti-DnaA antibodies. A range of amounts of purified His-DnaA [34] was used for quantification (only the 8.5 ng quantity is shown as an internal standard). Numbers listed below are amounts of DnaA in each sample calculated using densitometry.

We first localized DnaA within *E. coli* by determining its distribution over different cell fractions, repeating in general the experiment of Sekimizu *et al*., [39], but with a different disruption method and a more refined fractionation as described in Material and methods. The presence of DnaA in steady-state growing *E. coli* BL21 cells and in different cell fractions was detected by immunoblotting with further quantification using a range of amounts of purified His-tagged DnaA as a standard (Figure 1). After separation of cell lysate, the majority of the protein was found in the supernatant. However, DnaA was also found in the insoluble fraction, which corresponds to the envelope and which is usually designated as the total membrane fraction. After separation of membranes in sucrose gradient, most of DnaA was found in the inner membrane fraction (Figure 1). To calculate the relative amount of DnaA bound to the membrane we used the amount of the total protein, 15 and 1.3 mg, found in the soluble and the membrane fractions respectively. An equal amount of protein, 10 µg, from each fraction was loaded on the gel and then processed for immunoblotting. 2.4 and 2.9 ng of DnaA were revealed in this amount of soluble and membrane fractions, respectively (Figure 1). That means that the soluble fraction contained about 3.6 µg of DnaA overall, and the membrane fraction contained 0.38 µg, which represents about 10% of the total cellular content of the protein (in assumption that the partitioning is the same in all cells in the population). An alternative calculation, assuming that about 7.5% of total cellular protein is in the inner membrane [44], results in about 5% of the total DnaA associated with the inner membrane (using 2.7 ng of DnaA found in 10 µg of the inner membrane protein and 3.5 ng of DnaA in 10 µg of total cell lysate protein –2.7×7.5/3.5×100). These values underestimate the proportion of DnaA in the membrane since some DnaA must be lost during fractionation and recovery. Furthermore, DnaA is a peripheral protein and a poor retention to membrane may be expected during a harsh disruption in the French press and several dilutions.

### 2. Construction of mCherry Fusions with DnaA and its Fragments

**Figure 2:**
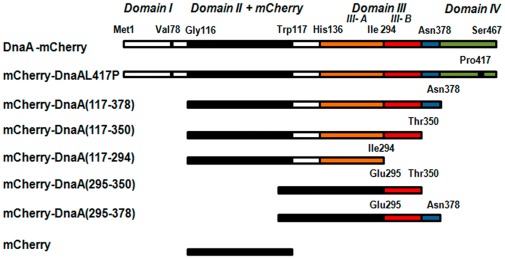
DnaA constructs used in this study. The constructs were engineered as described in Materials and Methods. Amino acids flanking DnaA domains and fragments are shown. Domains I and II are in white, mCherry sequence is colored black, domain III is represented by subdomain IIIa (orange) and IIIb (red) connected by the linker (blue) to domain IV (in green). Point mutation in domain IV, L417P, is also marked.

To construct DnaA-mCherry internal fusion protein we used the same strategy as described by the Crooke group [12], inserting the *mCherry* gene in the middle of *dnaA* domain II just after Gly116 (Figure 2). The hybrid gene, under the control of the arabinose promoter, was inserted into the moderate copy number plasmid pBAD24. DnaA-mCherry initiation activity was tested with Flow Cytometry, using the run-out procedure (see Material and methods). Expression of DnaA-mCherry caused an over-initiation of DNA replication in *E. coli*, similar to that observed upon expression of the wt DnaA (Figure S1). In addition, DnaA-mCherry over-expression led to cell filamentation and impaired cell growth, as previously observed with wt DnaA [45], [46], and also to aberrant partitioning of nucleoids (Figure S2). Furthermore, the plasmid-encoded DnaA-mCherry allowed growth of a DnaA^ts^ strain WM433 (*dnaA204*) at a non-permissive temperature, 42°C. We thus conclude that this construct confers a normal initiation activity *in vivo* and may be used further to localize DnaA.

**Figure 3:**
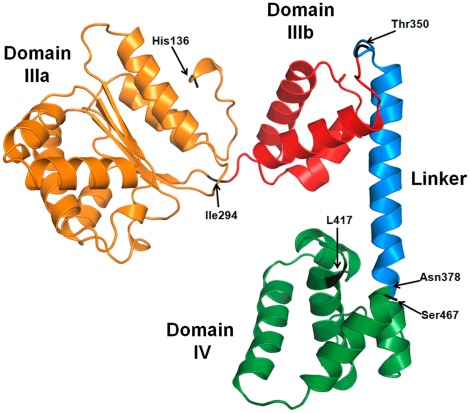
Predicted structure of *E. coli* DnaA Domains III and IV. The structure was obtained by threading the corresponding primary sequence on the structures of *Aquifex aeolicus* DnaA, DnaA Domain III of *Thermotoga maritima* and *E. coli* domain IV (Protein Data Bank accession codes 1L8Q, 2Z4R and 1J1V, respectively) using I-TASSER server (http://zhanglab.ccmb.med.umich.edu/I-TASSER/). In orange - Domain IIIa, containing the ATP-binding region of AAA^+^? ATPase-type proteins (Walker motif), in red - Domain IIIb, sensor region 2, in blue - linker segment that connects Domain III and IV, considered to have a membrane interaction function, and in green - the C-terminal Domain 4, DNA binding domain (for domains designation and function see [1]). Amino acid residues chosen as C-terminals of different domains and used to design DnaA fragments (Figure 2) are shown by arrows. Images were generated using Pymol software (www.pymol.org).

Construction of mCherry fusions with DnaA fragments was based on the predicted structure of *E. coli* DnaA. It was important to design carefully the borders of the fragments particularly in the region suspected to bind membrane (see below) in view of the low homology of the linker region with known structures. This was achieved by homology modeling using the I-TASSER server [47], [48], in which the *E. coli* DnaA sequence was threaded over all determined DnaA structures available, and the resulting structure is shown in Figure 3. The region of domain IIIb and, specifically, the linker helix appeared slightly different from the previously suggested structure [4], [49], but more similar to the determined structures [14], [50]. The structure in Figure 3 was therefore used and amino acids Ile294, Thr350 and Asn378 were chosen as the end-points of the fragments to avoid disturbing the native structure of functional domains.

### 3. Intracellular Distribution of mCherry Hybrids with DnaA

**Figure 4:**
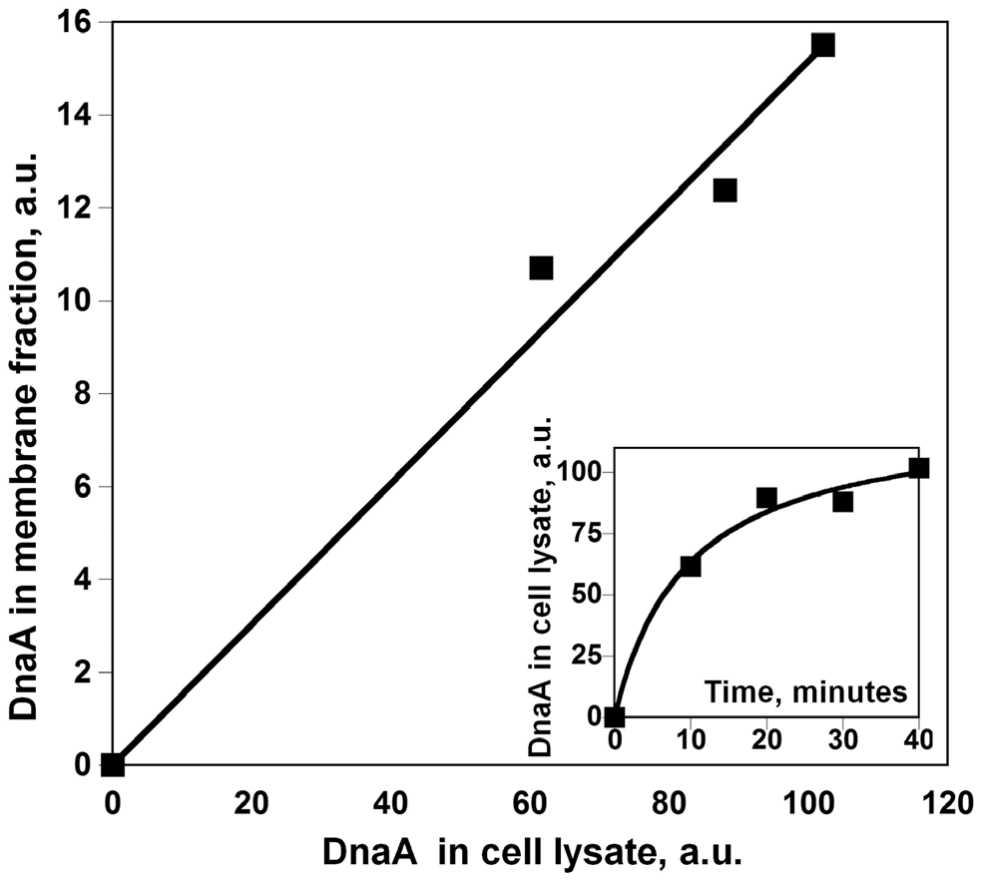
Increase of DnaA-mCherry content in the membrane fraction as a function of its expression level. *E. coli* BL21 harboring pBAD24(*dnaA-mCherry*) was induced by 0.2% arabinose for time periods shown on the graph in the inset. DnaA-mCherry content in cell fractions is presented in mCherry fluorescence intensity units, determined as described in Experimental procedures.

To verify that retention of DnaA-mCherry on the membrane reflects its total cellular content, we performed fractionation of BL21 cells after controlled moderate expression of DnaA-mCherry from the pBAD24 plasmid. Quantitation of the mCherry fluorescence intensity in the whole cell lysate and in the membrane fraction revealed that elevation of the total DnaA-mCherry concentration is accompanied by a comparative increase in the amount of DnaA-mCherry on the membrane, indicating that DnaA-mCherry does not saturate the membrane in the tested range of concentrations (Figure 4). Note, that the total concentration of DnaA-mCherry approaches the maximal level during 40 minutes of expression (Figure 4, inset), so that a longer expression does not significantly increase the protein level.

**Figure 5:**
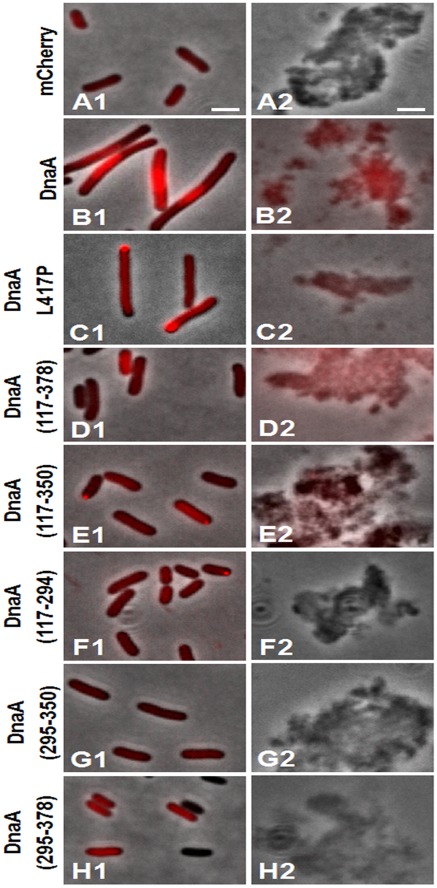
Combined phase-contrast and fluorescence images of *E. coli* BL21 expressing different constructs (see **Figure 2** **) of mCherry-DnaA (Left column) and inner membranes derived from corresponding cells (Right column).** Cells expressing mCherry alone are shown in line A. For an example of separate phase-contrast and fluorescence images see Figure S2. Inner membrane vesicles are about 120 nm in size and therefore are hardly visible in homogeneously dispersed sample. However, they tend to aggregate in suspension forming large clumps visible in phase contrast. Scale bar is 2 µm.

Microscopic examination of *E. coli* BL21 cells expressing mCherry or DnaA-mCherry demonstrated an even distribution of mCherry alone (Figure 5, frame A1) and a predominantly nucleoid localization of DnaA-mCherry in elongated cells with unsegregated nucleoid (Figure 5, frame B1). The foci or helical patterns of DnaA observed by others [12], [13] were not apparent. This difference may stem from the much higher expression level of our plasmid-born construct compared with native *dnaA*. Our fluorescent DnaA could not be localized with optical microscopy to the membrane despite its relatively high expression level. However, this is comprehensible given that we found only 5–10% of the native protein in the inner membrane fraction, which may be insufficient for detectable fluorescence contrast between the membrane and the cytoplasm.

**Figure 6:**
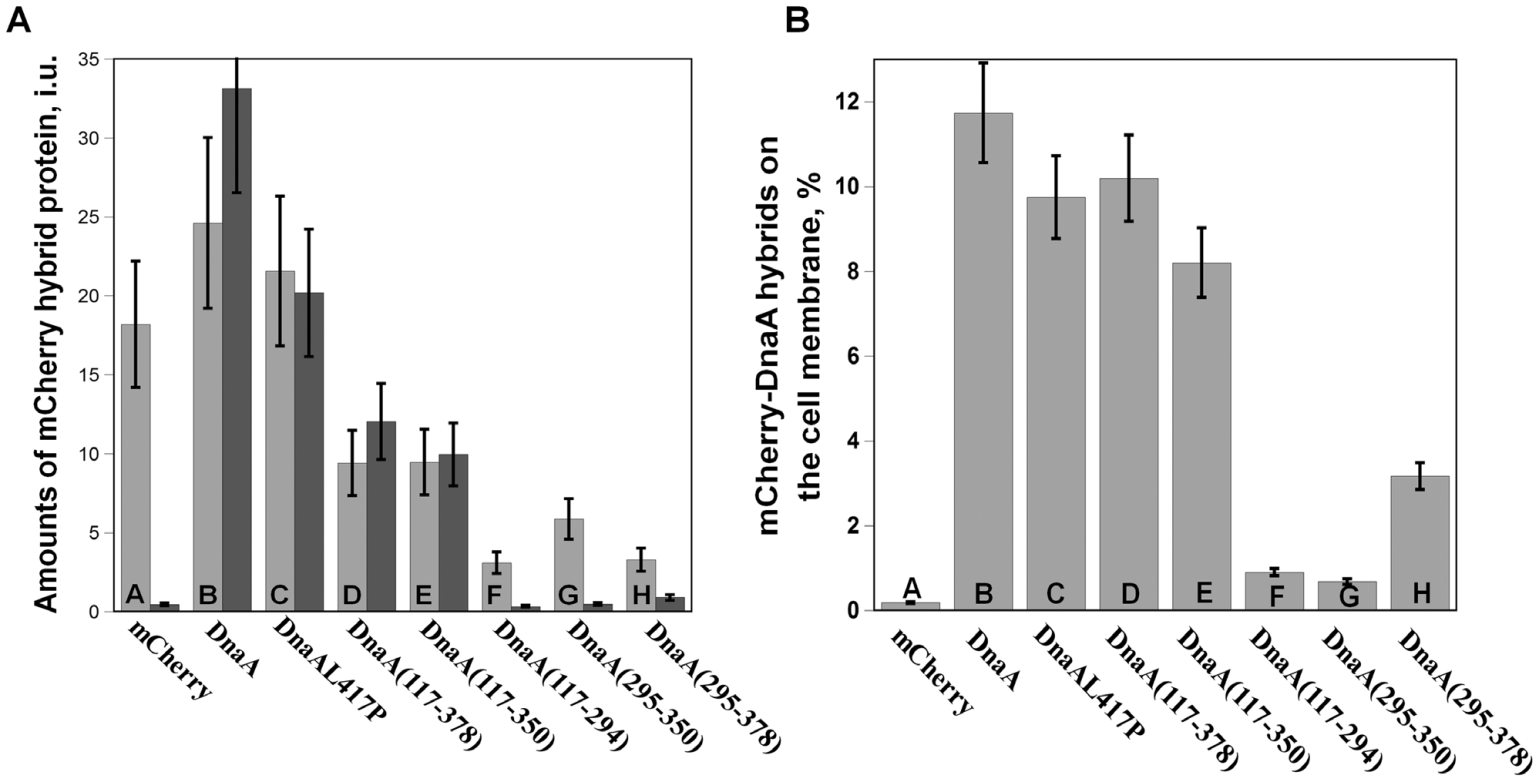
Intracellular distribution and membrane affinity of various mCherry-DnaA constructs. **Panel A.** Amounts of different mCherry-DnaA constructs in whole cell lysates (light columns) and in IMV (dark columns) depicted in fluorescence intensity units. The constructs were expressed in *E. coli* BL21 from pBAD24 by 0.2% arabinose for 2 hours. The cells were harvested, lyzed and fractionated as described in Experimental procedures. Fluorescence intensity was measured in cell lysate and in the IMV fractions brought to the same protein concentration (0.15 mg/ml). Error bars represent SE from three independent experiments. **Panel B.** Cellular membrane fraction of mCherry-DnaA constructs. Membrane retention of mCherry hybrid proteins on IMV has been calculated as intensity levels in IMV normalized by the steady-state level of the respective proteins. The latter was obtained from fluorescence intensities in cell lysates, taken as a measure for overall expression level, corrected for the extent of degradation of each construct (Figure S3), accounting for only the fraction of the full-size construct. The cellular membrane fraction of DnaA constructs has been calculated assuming that about 7.5% of total cellular protein is in the inner membrane [44] and shown as percent of total cellular mCherry-DnaA content. Error bars represent SE from three independent experiments.

The inner membrane vesicles (IMV) fraction obtained from cells expressing DnaA-mCherry appeared highly fluorescent visually (Figure 5, frame B2) and displayed a typical mCherry spectrum. Quantitation of the fluorescence intensities in the cell lysate and corresponding IMV (Figure 6A, column B) resulted in about 11% of total cellular fluorescence retained in the membrane fraction (Figure 6B, column B), perfectly consistent with our results on distribution of the native protein. Expression of mCherry alone resulted in non-fluorescent IMV (Figure 5, frame A2) and hardly detectable fluorescence intensity (Figure 6A, column A) that may be due to an occasional entrapment of mCherry during the cell disintegration.

The DnaA-binding capacity of the nucleoid is high relative to the total cellular DnaA content (see third paragraph in Discussion), so that the vast majority of native or even fusion proteins is bound to the nucleoid, leaving only a small amount of DnaA available for membrane binding. To obtain the expressed DnaA mainly in the cytoplasm and eventually more of it on the membrane, we exploited a L417P mutation in DnaA domain IV known to impair DNA binding [51]. Indeed, mCherry-DnaA L417P showed no nucleoid localization in slightly elongated cells and the protein was evenly dispersed inside the cells apart from an inclusion body at one or both poles (Figure 5, frame C1). Even under these conditions there was no distinctive membrane staining. Notably, the cytoplasm displayed about the same brightness as in the cells expressing native DnaA-mCherry (compare images B1 and C1 in Figure 5). Apparently, the solubility of DnaA in cytoplasm is limited, forcing the protein excess into inclusion bodies in the absence of DNA binding. It is also possible, that DNA binding impairment makes this mutant more susceptible to proteolysis (Figure S3).

Like the native DnaA-mCherry, mCherry-DnaA L417P was also well retained in the IMV fraction after cell fragmentation (Figure 5, frame C2) and showed a similar distribution to the membrane (Figure 6B, column C). A clear conclusion from this experiment is that association of majority of DnaA (at least 80%) with the membrane is direct and not a result of binding to membrane-associated fragments of DNA.

### 4. Intracellular Distribution of mCherry Hybrids with DnaA Fragments

**Figure 7:**
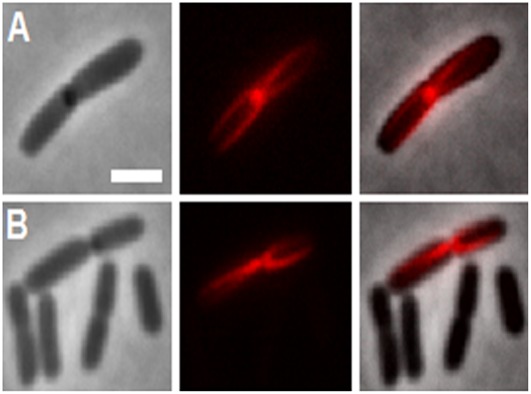
Fluorescent structures in *E. coli* BL21 overexpressing mCherry-DnaA(117–378). Two examples (A and B) of phase-contrast (left image), fluorescence (middle) and their overlay (right) images of BL21 cells expressing mCherry-DnaA(117–378) during 4 hours. The cells visible as non-fluorescent in B actually display a fluorescence level similar to those shown in Figure 5D1, that is just too low relative to intensity of inclusion bodies. Scale bar is 2 µm.

The segment of DnaA suspected to bind membrane is located at the end of domain IIIb adjacent to domain IV [1], based on *in vitro* experimental data [31], [32], [52]. We have examined membrane retention of *in vivo* expressed mCherry fusions with complete domain III and its fragments (Figure 2) chosen according to the predicted structure of *E. coli* DnaA (Figure 3). The complete domain III fusion construct, mCherry-DnaA(117–378), was expressed at a 2.5 fold lower level than the full-length DnaA induced at the same conditions (concentration of arabinose and induction time) (Figure 6A). Note also a high proteolytic stability of this construct (Figure S3). The expressing cells were evenly stained, while corresponding IMV were again highly fluorescent (Figure 5, frames D1 and D2), showing almost the same membrane partitioning as the wt fusion (Figure 6B). Further expression of this construct perturbed cell division in a small percentage of cells (∼10%), and this was accompanied by formation of inclusion bodies at the would-be division site (midcell) with “projecting rays” of fluorescent protein adjacent to the membrane on both sides of the cell (Figure 7). Notice that the rays emanating from inclusion bodies into two daughter cells seem to be oriented to different planes. This unusual shape of inclusion bodies is reproducible and was also observed when this construct was expressed in different *E. coli* strains; it was never observed with other constructs examined in this work, and we are not aware of any similar observations in the literature.

It is commonly accepted that the segment between residues 372–381 comprises a functional domain of DnaA responsible for the membrane binding [1] based on differences in membrane sensitivity between tryptic and chymotryptic fragments of DnaA [28]. Photocrosslinking results suggested that a larger region between residues 309–399 may be inserted into the membrane [29], [31], [32], [52]. In our predicted structure, these regions correspond to α-helixes 299–312, 319–327, 334–346 and 356–376. We therefore tested the membrane binding property of domain III truncated at residue 350.

mCherry-DnaA(117–350) was visible in the cytoplasm, being evenly distributed along the cell and in some cases forming an inclusion body at one of the cells poles (Figure 5, frame E1). It was very similar to mCherry-DnaA(117–378) in the expression level and with the extent of membrane partitioning slightly lower than wt (Figure 6A and B, columns E).

Further truncation to residue 294, removing the whole domain IIIb, resulted in even lower expression of mCherry-DnaA(117–294) (Figure 6A, column F), the same cellular distribution as the previous fragment (Figure 5, frame F1), but almost a complete loss of membrane retention (Figure 5, frame F2, Figure 6B, column F). This result indicates that the three α-helixes comprising domain IIIb might play an essential role in membrane binding. Surprising enough, this segment alone, when fused to mCherry (mCherry-DnaA(295–350)) and expressed as other constructs, was barely retained on IMV (Figure 5, frame G2, Figure 6B, column G). However, adding another α-helix, 356–376, to the preceding three helices to give mCherry-DnaA(295–378) remarkably increased the membrane retention (Figure 5, frame H2, Figure 6B, column H). It would seem that the properties of the complete functional domain are not merely the sum of its separate parts. Rather, the membrane binding capability may be composed of a harmonized interaction of different parts of the protein with the membrane, with each of these parts contributing to the correct structure of the whole.

## Discussion

### A Small but Considerable Amount of DnaA is Located to the Membrane

Several precautions must be taken when using cell fractionation to localize a membrane peripheral protein – as opposed to an integral membrane protein – within the cell. On the one hand, nonspecific and third-party mediated binding, particularly with such multifunctional protein like DnaA, must be considered. On the other hand, the reversibility of binding should be taken into account. Our immunoblotting of native DnaA in different cell fractions revealed small but reliable amounts of the protein in the inner membrane fraction (Figure 1). Other experiments were performed with a fluorescent derivative of DnaA, allowing better detection and quantification in combination with a set of mutants and DnaA fragments. The presence of DnaA in the IMV fraction is obviously not a result of nonspecific binding or entrapment in vesicles since mCherry alone does not appear in this fraction at all (Figure 5, frame A2, Figure 6, column A). This presence is not mediated by membrane-associated DNA fragments since a DnaA mutant deficient in DNA binding is similarly present in the IMV fractions (Figure 5, frame C2, Figure 6, column C). Elimination of the DnaA N-terminus, responsible for oligomerization [8], rules out incorporation of the fluorescent derivative into existing complexes (aggregates) of native protein associated with the membrane. The effective membrane retention of the domain III lacking both terminal domains, mCherry-DnaA(117–378) (Figure 6, column D), confirms that this fragment binds to the membrane by itself without the need for an intermediary.

Our estimation, with both native and fluorescently tagged protein, of about 10% of total cellular content of DnaA bound to the membrane in *E. coli* is consistent with the 20% assessment first reported by Sekimizu and Kornberg, 1988 [39]. The *in situ* counting of immunogold particles [42] revealed about 70% of DnaA adjacent to the membrane. The method however does not distinguish between nucleoid-associated DnaA and that directly bound to the membrane of the fixed cells. In two other bacterial species, *H. pylori* and *M. tuberculosis*, where the fractionation method was used to localize DnaA [40], [41], the authors claimed that the majority of DnaA is on the membrane. This conclusion was based on the similar amounts of DnaA they found in whole cell lysate and in the membrane fraction. However, the quantities of total protein in the lysate and membrane fraction loaded on SDS-PAGE were the same and their estimate did not take into account the fact that membrane proteins represent at most 10% of the total cellular protein content in bacteria. Therefore, it follows from the results obtained with *H. pylori* and *M. tuberculosis* that only about 10% of cellular DnaA is associated with the membrane. Our results lead to the conclusion that a small but significant amount of DnaA is located on the membrane in live cells. It should be noted that this estimate is a population average with respect to the cell cycle and may vary both during the cycle and as a function of growth rate. A brief period when the level of DnaA bound to membrane is high would seem possible in view of the high binding capacity of the membrane for DnaA, found in our experiments when expression of DnaA was varied (Figure 4).

Is this small amount of membrane-associated DnaA understandable in terms of cell function and structure, and in particular compartmentalization? The number of DnaA molecules per individual *E. coli* is known to vary according to growth rate, the state of the culture and the nature of the strain [39], [53], [54]. However, this number appears constant per *oriC* independently of growth rate and for *E. coli* B/r it is about 350 DnaA molecules [54]. At high growth rates, the total number of DnaA can reach 2500 molecules per cell. The DnaA binding capacity of the chromosome is represented by about 300 DnaA boxes with differing affinities [55], including two regions with the highest affinity - *oriC* and *datA*. The last two are able to bind 45 and 370 DnaA molecules with apparent dissociation constants of 8.6×10^−9^ M and 1.7×10^−8^ M respectively [56]. Not surprisingly, these regions bind the vast majority of DnaA in the cell and appear as bright foci when a fluorescent derivative of DnaA is expressed to the native level [13]. Moreover, comparing the total cellular DnaA content with the *datA* binding capacity and taking into account the number of *oriC* per cell and almost one-to-one *oriC* and *datA* stoichiometry (based on their close proximity on the chromosome) leads to the conclusion that the number of free, cytoplasmic DnaA may be quite low. Indeed, a moderate increase in *datA* dosage delayed initiation apparently by titration of free DnaA [57]. The affinity of DnaA for phosphatidylglycerol membrane is about 10^−7^ M [34], which is considerably lower than that for *datA*. Our estimate of membrane-bound DnaA as 10% of the total DnaA therefore appears plausible.

This distribution of DnaA in the cell explains why membrane localization of fluorescent derivatives of the protein was not revealed: just 10% of total fluorescence from the membrane is not expected to provide a detectable contrast in the cell image on the background of cytoplasmic and chromosomal fluorescence. In other words, at this intracellular distribution, optical resolution limits render membrane-bound DnaA practically invisible.

Our plasmid-borne DnaA-mCherry hybrid, expressed to levels higher than the native DnaA, stains the entire nucleoid, presumably occupying all its low-affinity binding sites as well, but still without visible membrane staining (Figure 5, frame B1). It is possible that this higher expression is the reason that we have not observed a helical pattern [12] or foci [13] since an excess of fluorescent DnaA may hide fine structures. Our goal however was to concentrate on membrane localization as an addition to nucleoid-associated patterns.

Our attempts to abolish DnaA-binding to DNA either by L417P mutation or by truncation of domain IV (so as to make the whole protein pool available for membrane binding) were not effective in localizing the constructs to the membrane (Figure 5, frames C1 and D1). In the case of the L417P mutant, in contrast to expression of the whole protein, a strong tendency to form inclusion bodies was observed (Figure 5, frame C1), while the DnaA(117–378) construct expression level was about 2.5 times lower than wt DnaA (Figure 6A). Thus, in both cases concentration of free DnaA was not actually elevated and with the relatively weak membrane binding affinity this failed to increase the staining contrast. Fortunately, cell fractionation was effective in separation from the overwhelming background revealing evident membrane retention (Figure 5, frames C2 and D2, Figure 6).

### In Search of a Putative Membrane Targeting Sequence of DnaA

The successful cell fractionation approach allowed us to further inspect different sections of DnaA for the membrane binding function. We focused on the DnaA(117–378) fragment that maintained the same membrane partitioning ability as the full length protein (Figure 6B, column D). According to our predicted structure for *E. coli* DnaA (Figure 3), this fragment is comprised of three apparently distinctive sections: domains IIIa and IIIb and the linker, corresponding to the solved homologous structure [2], [14]. The suggested membrane binding function was previously ascribed to the linker section, as mentioned in the Section 4 of Results. Truncation of this linker decreased the membrane retention only slightly (Figure 6B, column E). Further deletion of the entire domain IIIb, also suspected as interacting with phospholipids [31], [32], [52], led to a drastic loss of binding (Figure 6B, column F). This could point to domain IIIb as the major carrier of membrane binding function, but controversially, this domain alone showed very poor membrane retention (Figure 6B, column G). An improvement of retention by addition of the linker section to domain IIIb (Figure 6B, column H) may be attributable to stabilization and/or shaping of the resulting fragment structure. Still, the low extent of binding does not allow ascribing the entire binding function to this fragment. We thus conclude that fragment DnaA(295–378) is necessary for DnaA-membrane binding but by itself is not sufficient.

It could be, alternatively, that the DnaA(117–294) fragment might have lost the ATP binding capability and therefore is unable to bind to the membrane because of e.g. inability to form dimers/oligomers. However, we are not aware of any study demonstrating dependence of membrane binding of DnaA on the nucleotide. As mentioned by others [29], [58] and in our hands [34], the binding did not depend on the nucleotide type, ATP, ADP and even MANT-ATP. In fact, binding to the membrane drastically decreases the affinity for nucleotides so that the membrane-bound protein is essentially nucleotide-free. We therefore consider this explanation of the lower membrane affinity of fragments unable to bind ATP as implausible.

**Figure 8:**
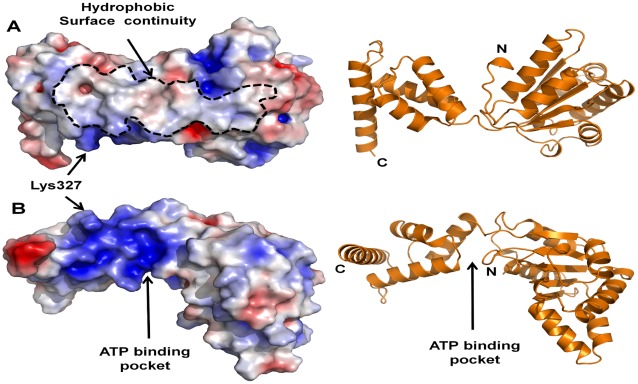
Electrostatic surface (left) and ribbon representation (right) of Domain III of predicted *E. coli* DnaA structure (**Figure 3**). The electrostatic surface was calculated by PyMol-molecular visualization system (red denotes negatively charged and blue denotes positive charge). **A.** Overview of Domain III, outlined is the apparent hydrophobic surface continuity extending throughout the “back” of the domain. **B.** A 90° rotation view, in which the ATP pocket is visible. The bulgy and positively charged Lys327 is shown by short arrows.

A well-known mode of reversible membrane association of many peripheral proteins is interaction through an amphiphylic α-helix [59], [60] that was searched previously also in DnaA. The four α-helixes comprising DnaA(295–378) are indeed amphiphilic, but this property seems to be essential for the intrinsic structure of the fragment rather than serving as a stand-alone membrane binding sequence (Figure 3). Alternatively, some proteins interact with the membrane through a surface area integrating both hydrophobic and polar groups belonging to different regions of the protein (e.g. [61]). Our finding that neither part of the whole DnaA(117–378), domain IIIa (117–294) or domain IIIb plus linker (295–378), provides the necessary binding affinity, led us to consider the surface properties of DnaA. An electrostatic surface model of the DnaA(117–378) fragment reveals a hydrophobic continuity extending throughout the back of domains IIIa and IIIb, opposite to the nucleotide-binding pocket (Figure 8A). This continuity is obviously composed of a number of hydrophobic residues from both domains. In domain IIIb, the hydrophobic patch is flanked by a positively charged bulge, originating mainly from the Lys327 residue, presumably responsible for the primary electrostatic interaction with anionic phospholipids. If binding to the membrane is indeed ensured by cooperative interaction of the mentioned surface as a whole, than dissection of this DnaA fragment into smaller parts, domain IIIa and IIIb, will eliminate membrane binding of each of them because of an insufficient energy of interaction. Our membrane retention results obtained with various fragments (Figure 6) strongly support this mechanism of binding through a concerted interaction of distant residues forming a surface, rather than by an individual helix.

**Figure 9:**
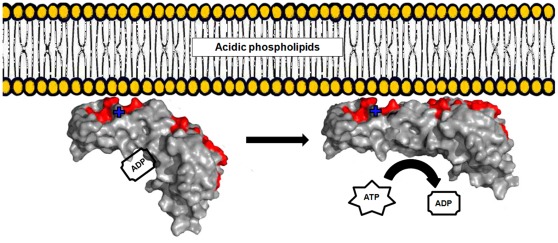
Cartoon illustrating binding of DnaA to the membrane surface. The primary electrostatic attraction of Lys327 (marked by **+**) to acidic phospholipids promotes hydrophobic interaction with the membrane of domain IIIb first, further enforced by interaction of domain IIIa (residues forming the contact hydrophobic continuity are colored red). The concerted interaction drives a conformational change in the protein, decreasing the affinity to the nucleotide. ADP is replaced for ATP due to a much higher concentration of the latter in a functional cell.

Moreover, the hydrophobic surface is convex (Figure 8B) relative to the membrane plane, so that the surface binding will most probably force a conformational change, straightening the protein and thus “lifting the lid” (domain IIIb) of the ATP pocket. We hypothesize that this binding-bending mechanism may explain how DnaA-membrane interaction triggers nucleotide exchange (Figure 9). Such conformational change induced by the membrane binding event is common for a range of so-called amphitropic proteins, activity of which is regulated via relocation from the cytoplasmic compartment to the membrane (see [60] for a review).

### Concluding Remarks and Hypothesis

Our results obtained both with native and with fluorescently tagged protein demonstrate that about 10% of cellular DnaA is located on the membrane in steady-state growing *E. coli*. We provide an explanation why this small fraction might be physiologically significant as representing the free DnaA available for initiation, opposed to the vast majority bound to the *datA* reservoir. We suggest that membrane binding is ensured by cooperative interaction of amino acid residues transversing the entire DnaA domain III. How do these findings and our binding-bending hypothesis contribute to our comprehension of bacterial cell cycle regulation?

In bacteria, the chromosome replication cycle is tightly coupled to growth rate in the sense that the initiation frequency of the discontinuous replication process is precisely adjusted to continuous process of growth, which is highly variable. The key regulatory mechanism of initiation is an increase in the DnaA-ATP form at the appropriate cell age and conversion back to the ADP-bound after the event [22]. Thus, DnaA acts as a molecular switch, in which the nucleotide recycling couples key processes in the cell. The relation between the *de novo* synthesis of DnaA (likely in ATP form), RIDA-induced ATP hydrolysis by DnaA and its reactivation is still unclear and requires further investigation. While ATP hydrolysis by DnaA is stimulated by RIDA only, the ATP recharging function was ascribed to the membrane and, later, also to DARS. Existence of more than one regulatory mechanism for such a crucial event does not seem superfluous. Possible interplay between the two regulatory mechanisms – membrane-associated and DARS has been extensively discussed [20]. We consider DnaA-membrane binding as a mediator of the functional state of bacteria, specifically, growth rate; this may help explain the enigmatic ‘initiation mass’ [62]–[64]. Indeed, we have suggested previously that the ‘initiation mass’ may result from the phenomenon of a highly cooperative inter-conversion between two functional states of DnaA driven by its membrane surface occupancy [34], [36]. Our present results provide a strong basis for extrapolation of this phenomenon to *in vivo* situation. Combined with the growing appreciation of the existence of membrane domains and their essential role in spatial and temporal organization of bacterial cell [37], [38], [65], this may stimulate new approaches and comprehension of fundamental regulatory mechanisms in bacteria.

## Materials and Methods

### Materials

Unless otherwise stated, reagent grade chemicals were from Sigma Chemicals, growth media components were from BD Biosciences and fluorescent dyes were from Molecular Probes (Invitrogen).

### Strains and Plasmids

*E. coli* strains used in this work were: **XL1-MRF** for genetic engineering, **Wm433** (Leu19, pro19, trp25, his47, thyA59, arg28, met55, deoB23, lac11, gall1, strA56, sulI, hsdSK12, dnaA204) for complementation and rescue of growth assay and **BL21(DE3) (**F– ompT gal dcm lon hsdSB(rB- mB-) λ(DE3 [lacI lacUV5-T7 gene 1 ind1 sam7 nin5]) for expression of DnaA constructs from pBAD24 and cell fractionation experiments. This strain, not containing any plasmids, was used also in experiments aimed to reveal the intracellular distribution of the native, chromosomal DnaA.

### Genetic Engineering

All genetic work and manipulations were performed on the basis of ***dnaA*** gene kindly provided by Prof. Elliott Crooke (Georgetown University, Washington, DC). All hybrid genes were inserted into pBAD24 vector by restriction with NdeI in front of the gene, and HindIII at the end, and consequent ligation of vector and insert. *E. coli* XL1-MRF was transformed with ligation products by electroporation and plated on LB-agar plates containing 0.2% glucose and 50 µg ml^−1^ ampicillin. Colonies were grown, plasmid purified and sequenced. *E. coli* BL21 were then transformed with the plasmids for physiological experiments. **DnaA-mCherry** fusion was prepared in two steps: first, an insertion of two consequent restriction sites KpnI and XhoI to the ***dnaA*** gene, immediately after Trp117, using 5′ primer overhang technique, and second, a PCR fragment containing *mCherry* flanked by the same two restriction sites was cut together with the new ***dnaA*** vector and ligated to create an ***mCherry–dnaA*** hybrid. **mCherry-DnaA L417P** point mutant was prepared by PCR using the primer-extension technique, with two primers carrying the mutation and the ***mCherry–dnaA*** hybrid as the template. The fragment was digested and inserted as described above. **mCherry-DnaA(117–378, 117–350 and 117–294)** were created by using PCR and 5′ primer overhang technique: forward primer that introduced an NdeI restriction site before the first Met of *mCherry* and a reverse primer containing stop codon and HindIII site after Asn378, Thr350 and Ile294, accordingly. The fragment was digested and inserted as described above. **mCherry-DnaA(295–378 and 295–350)** were build using PCR 5′ primer overhang: a forward primer that introduced an XhoI restriction site before Glu295 on the according segments above, then digested and inserted to the plasmid. For the full information on primers used in this work see Table S1.

### Temperature-sensitive Rescue Assay

Wm433 (*dnaA204*) [66] cells were transformed with pBAD24 bearing *dnaA*, *dnaA- mCherry* or empty plasmids. Cells were plated onto LB agar (50 mg ml^−1^ ampicillin) and cultured at 30°C. Transformants were streaked onto ampicillin plates with and without 0.2% arabinose and grown at 42°C for 12 hours. Colonies of Wm433 (*dnaA204*) bearing *dnaA* and *dnaA- mCherry* appeared only on arabinose-supplemented plates, confirming that plasmid-born DnaA and DnaA-mCherry are able to support growth of the temperature-sensitive *dnaA204* cells at 42°C. The transformants containing just the empty plasmid were not rescued by arabinose.

### Flow Cytometry

The number of *oriC* per cell was determined by flow cytometry as the number of chromosomes per cell following a run-out procedure of ongoing replications [64]. Cells were grown in LB medium at 37°C for 10 generations. L-arabinose was added for induction of DnaA and incubated for 50 minutes. Cells were then treated with 180 µg ml^−1^ rifampicin and 15 µg ml^−1^ cephalexin for approximately 5 doubling times. The cells were fixed 70% ice-could ethanol and stored at 4°C. Samples were washed and resuspended in a Tris 10 mM, MgSO_4_ 20 mM buffer (pH 7.5) and diluted to OD_450_ = 0.015. SytoxGreen, for DNA labeling, was added to final concentration of 1.5 µM and samples were read in a FACSCalibur (Beckton Dickinson) Flow cytometer.

### Cell Growth and DnaA-mCherry Induction

BL21 cells carrying pBAD24 encoding various DnaA-mCherry constructs were grown overnight in LB medium supplemented with ampicillin (50 µg ml^−1^) at 37°C, diluted 1000-fold in fresh medium and grown at steady-state to OD_600_ = 0.15. At this stage, arabinose was added to final concentration of 0.2% and induction continued for two more hours. At the end of induction period, chloramphenicol (100 µg ml^−1^) was added to stop protein synthesis and incubation continued for 30 min at 37°C and additional 30 min on ice to ensure full maturation of the fluorescent protein. Samples were then taken for fluorescence microscopy, fluorescence intensity measurements and for cell fractionation.

### Fluorescence Microscopy, Sample Preparation

Object slides were coated with a flat layer of 1% agarose gel in DPBS (GIBCO). 4 µl of cells or of IMV were immobilized on the coated slides and visualized using a Nikon Digital Sight DS-U1 cooled CCD camera mounted on an Nikon inverted fluorescence microscope (Nikon Eclipse TE2000-S, Badhoevedorp, The Netherlands) equipped with a Plan Fluor 1003 oil NA1.3 objective (Nikon). The fluorescence was excited and detected with a 100 W mercury lamp in combination with a DiI filter (Chroma Technology; exciter D540/25 nm, emitter D605/55). Images were obtained and processed using the microscope domain program NIS-Elements Basic Research (Nikon Instruments, Melville, NY). To enable comparing of amount of fluorescence in different samples, the images were acquired at standard excitation intensity, exposure and gain, chosen in manual mode. When DNA visualization was desired, cells were stained with DAPI (2 µM).

### Florescence Intensity of mCherry-hybrids

Fluorescence emission spectra of mCherry were measured on a Perkin-Elmer LS55 fluorimeter (Perkin-Elmer, Beaconsfield, England) at 25 °C, at excitation wavelength of 550 nm, slits width of 5 and 5 nm for excitation and emission (Perkin-Elmer, Beaconsfield, England). BL21 cells were concentrated to OD_600_ = 1, washed with PBS and lysed in 1 ml of dissolving buffer (6 M Urea, 0.5% SDS, 300 mM NaCl and 50 mM of NaH_2_PO_4_, PH = 8.5) for 30 min at room temperature. Samples were centrifuged for 30 min at 13,000×g and supernatant was examined in fluorimeter. For mCherry intensity in IMV, 100 µg of total IMV protein (measured by Bradford) was added to 1 ml of dissolving buffer and last step was repeated. Background signal from non-expressing cells and respective IMV was subtracted from all fluorescence measurements.

### Cell Fractionation

Cells were fractionated by the method of Osborn *et al.* [67] with minor modifications. Briefly, after protein induction cells were harvested by centrifugation and washed with cold Tris buffer (Tris-HCl 0.05 M pH 7.5) and re-suspended in cold Tris-sucrose buffer (Tris-HCL 0.25 M, sucrose 0.05 M, pH 7.5) supplemented with DTT 1 mM and PMSF 0.375 mM, 10 ml of buffer for 0.5 liter of cell culture. The cells were broken by passing the suspension twice through a French press (FA-032, Thermo-Electron), at 10,000 psi. After each cycle an extra 1 mM of DTT was added. Cell debris was removed by centrifuging at low speed (6000×g) and the supernatant was centrifuged at 165,000×g for 90 min (SORVALL Discovery 90SE, BECKMAN 70.1 Ti rotor). Pellet was gently suspended in 1 ml of Tris-sucrose buffer and mounted on the top of discontinuous sucrose gradient, composed of sucrose layers of 55, 50, 45, 40, 35 and 30% in Tris buffer. Sucrose gradient was centrifuged at 170,000×g for 14 hours (SORVALL Discovery 90SE, BECKMAN SW-40 rotor). Outer membranes were collected from the bottom of the gradient [67]. Inner membranes, appeared as a distinct brown band in the 35% sucrose layer, were collected and centrifuged again at 165,000×g for 90 min (SORVALL Discovery 90SE, BECKMAN 70.1 Ti rotor). Membranes were re-suspended in cold Tris-sucrose buffer, frozen in liquid nitrogen and stored at −80°C. The protein content in membrane preparations were attained by Bradford method. The quality of IMVs was verified using measurement of NADH-oxidase activity [68].

### DnaA Immunoblotting

Samples of 10 µg of total protein were denatured in sample buffer and loaded on 8.5% acrilamid SDS gel. Following electrophoresis and transfer the nitrocellulose membrane was reacted with 1∶20,000 anti-DnaA antibody serum and progressed to reaction with goat anti-rabbit HRP antibodies (KPL). Chemiluminescence was then measured by Fujifilm LAS-3000 camera, band intensities were analyzed with ImageJ software. The same immunoblotting performed with 1–15 ng of purified His-DnaA loaded on SDS-PAGE showed a good linear correlation with the amount of protein (not shown).

### Estimation of Degree of Degradation of Expressed Proteins

Possible degradation products of the expressed mCherry-DnaA constructs were detected using an antibody against mCherry. We have exploited the fact that fluorescent proteins are known for their high proteolytic stability and thus can be used as a marker for proteolysis of their fusions. Different constructs were expressed as described above, and the total cellular proteins were subjected to SDS-PAGE and Western blotting. The blot was exposed to 1∶1000 monoclonal, affinity-purified anti-mCherry antibodies (Clonetech) and proceeded to reaction with secondary goat anti-mouse HRP antibodies. Chemiluminescence was measured as described above and band intensities were quantified using the ImageJ profile routine. Intensity profiles were analyzed using PeakFit program (version 3.18, Jandel Scientific, San Rafael, CA) for determining the amount of protein in each band. In assumption that only the full construct is capable (or not) for membrane binding, we have corrected the results of binding to the fraction of the full construct (Figure 6B).

## Supporting Information

Figure S1 Over-initiation of DNA replication caused by expression of plasmid-born wt DnaA or DnaA-mCherry. Flow cytometry plots of DnaA-expressing *E. coli* BL21cells. Average number of chromosome equivalents in non-expressing cells is 5.8, and 7 and 6.6 in DnaA and DnaA-mCherry-expressing, respectively. Cells were grown in LB medium (37°C), induced with 100 µM arabinose for 50 minutes, and then treated with rifampicin and cephalexin prior to analysis (see Experimental procedures).
(TIF)

Figure S2 Phase-contrast (A,D), fluorescence (B,E and G) and overlaid (C, F and H) images of *E. coli* BL21 cells expressing wt DnaA (A-C) and DnaA-mCherry (D-H). Image C is the overlay of A and B, Image F is the overlay of D and E and Image H is the overlay of D and G. Nucleoids stained with DAPI are colored in blue and mCherry fluorescence is in red. For details of staining and microscopy see Experimental procedures. Scale bar is 2 µm.
(TIF)

Figure S3 Immunoblot of *E. coli* BL21 lysates following induction of different mCherry constructs (see Scheme 1) from pBAD24 with 0.2% arabinose for 2 hours. The cells were harvested, lyzed in sample buffer, and subjected to SDS-PAGE (30 µg of protein loaded per lane), western-blotted and exposed to monoclonal, affinity-purified anti-mCherry antibodies. After staining the blot with secondary antibodies, the band intensities were quantified using the ImageJ profile routine and the profiles analyzed using the PeakFit program (version 3.18, Jandel Scientific, San Rafael, CA). The numbers below each lane represent the fraction of the full-size construct after taking the degradation products into account. Molecular weights of the constructs based on their sequences are shown on the left side.
(TIF)

Table S1 Primers used in this work for construction of the various mCherry-DnaA constructs shown in Figure 2.
(TIF)
