# Supplementary figures and images for: Association of the Chromosome Replication Initiator DnaA with the Escherichia coli Inner Membrane In Vivo: Quantity and Mode of Binding
Source: PLoS One. 2012 May 4;7(5):e36441. doi: 10.1371/journal.pone.0036441 (PMC3344877; doi:10.1371/journal.pone.0036441)

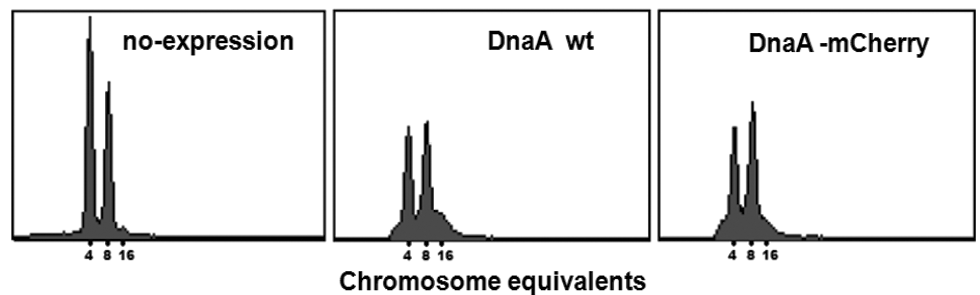

Supplement: Figure S1 — Over-initiation of DNA replication caused by expression of plasmid-born wt DnaA or DnaA-mCherry. Flow cytometry plots of DnaA-expressing E. coli BL21cells. Average number of chromosome equivalents in non-expressing cells is 5.8, and 7 and 6.6 in DnaA and DnaA-mCherry-expressing, respectively. Cells were grown in LB medium (37°C), induced with 100 µM arabinose for 50 minutes, and then treated with rifampicin and cephalexin prior to analysis (see Experimental procedures). (TIF) [file pone.0036441.s001.tif]

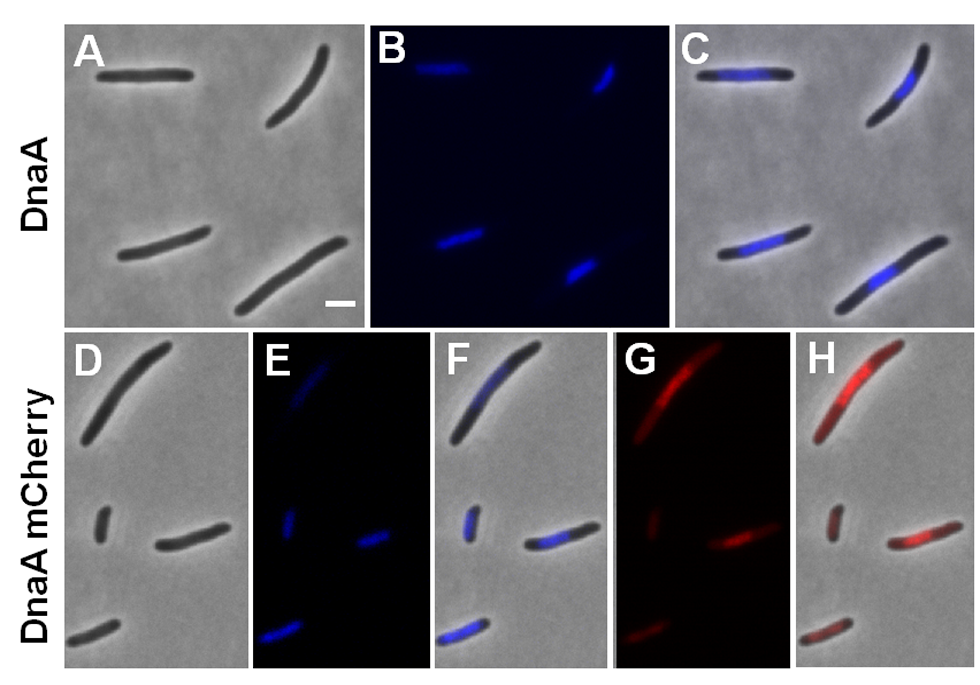

Supplement: Figure S2 — Phase-contrast (A,D), fluorescence (B,E and G) and overlaid (C, F and H) images of E. coli BL21 cells expressing wt DnaA (A-C) and DnaA-mCherry (D-H). Image C is the overlay of A and B, Image F is the overlay of D and E and Image H is the overlay of D and G. Nucleoids stained with DAPI are colored in blue and mCherry fluorescence is in red. For details of staining and microscopy see Experimental procedures. Scale bar is 2 µm. (TIF) [file pone.0036441.s002.tif]

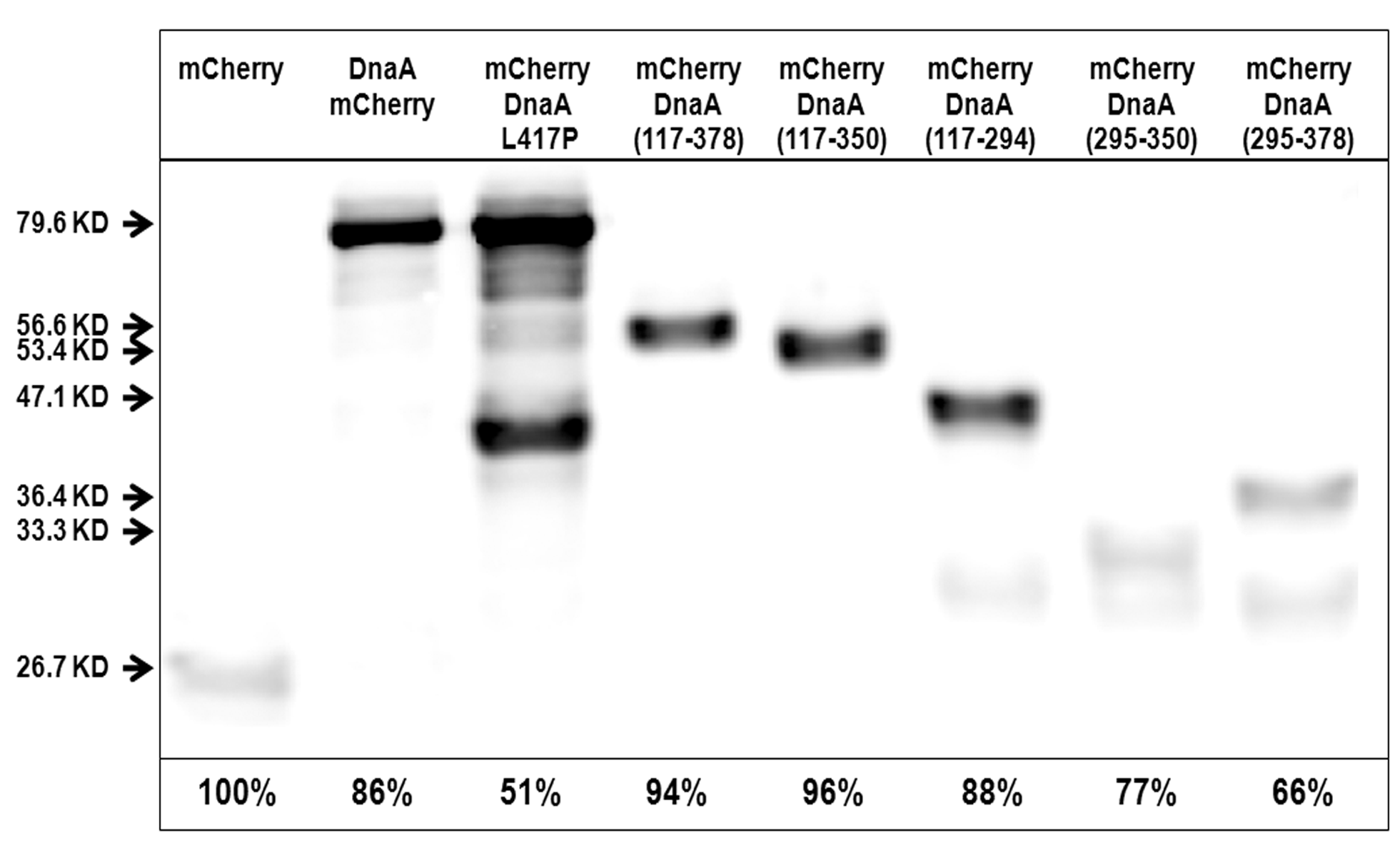

Supplement: Figure S3 — Immunoblot of E. coli BL21 lysates following induction of different mCherry constructs (see Scheme 1) from pBAD24 with 0.2% arabinose for 2 hours. The cells were harvested, lyzed in sample buffer, and subjected to SDS-PAGE (30 µg of protein loaded per lane), western-blotted and exposed to monoclonal, affinity-purified anti-mCherry antibodies. After staining the blot with secondary antibodies, the band intensities were quantified using the ImageJ profile routine and the profiles analyzed using the PeakFit program (version 3.18, Jandel Scientific, San Rafael, CA). The numbers below each lane represent the fraction of the full-size construct after taking the degradation products into account. Molecular weights of the constructs based on their sequences are shown on the left side. (TIF) [file pone.0036441.s003.tif]

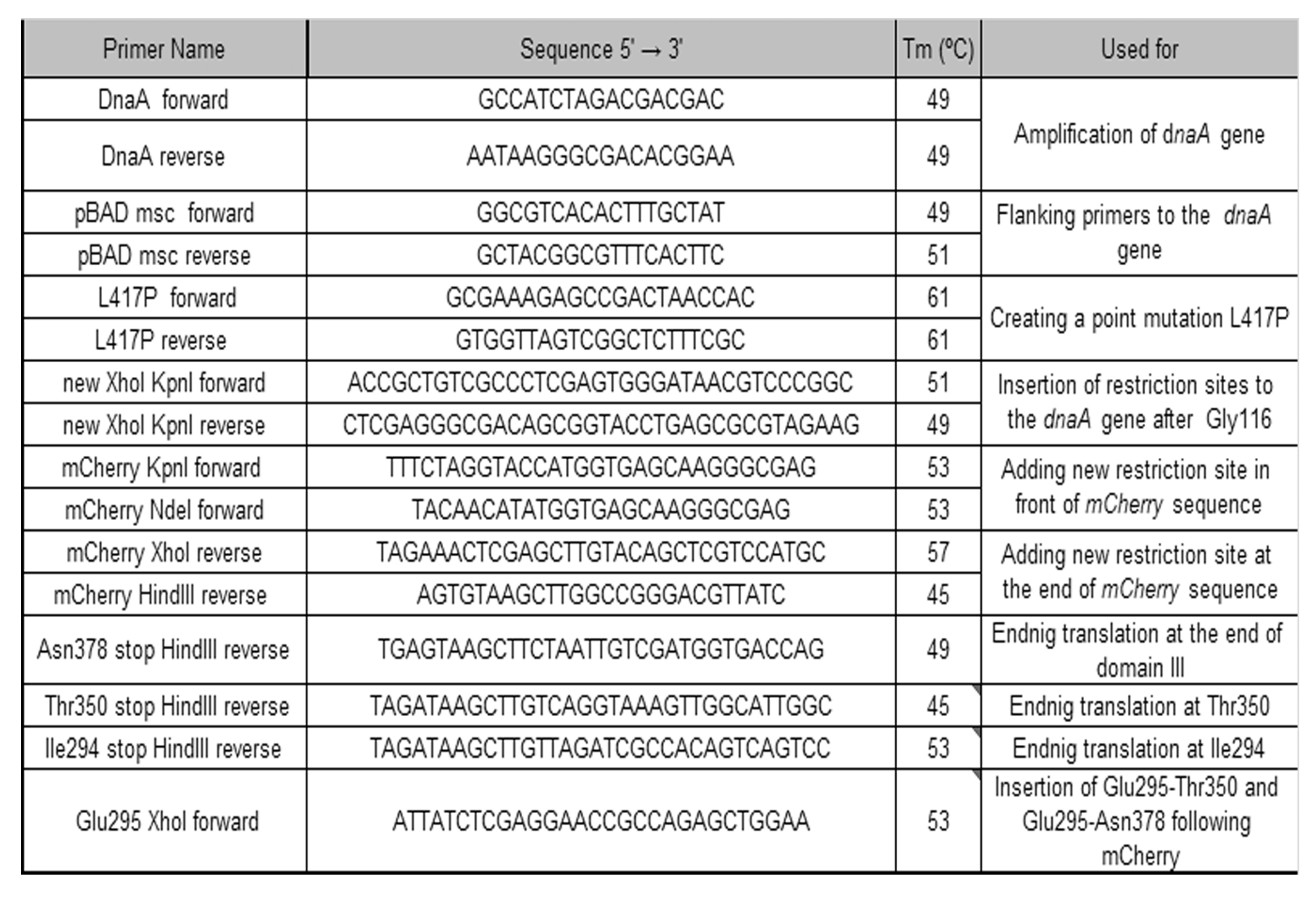

Supplement: Table S1 — Primers used in this work for construction of the various mCherry-DnaA constructs shown in Figure 2. (TIF) [file pone.0036441.s004.tif]
